# Supplementary figures and images for: Identifying cellular RNA-binding proteins during infection uncovers a role for MKRN2 in influenza mRNA trafficking
Source: PLoS Pathog. 2024 May 16;20(5):e1012231. doi: 10.1371/journal.ppat.1012231 (PMC11135703; doi:10.1371/journal.ppat.1012231)

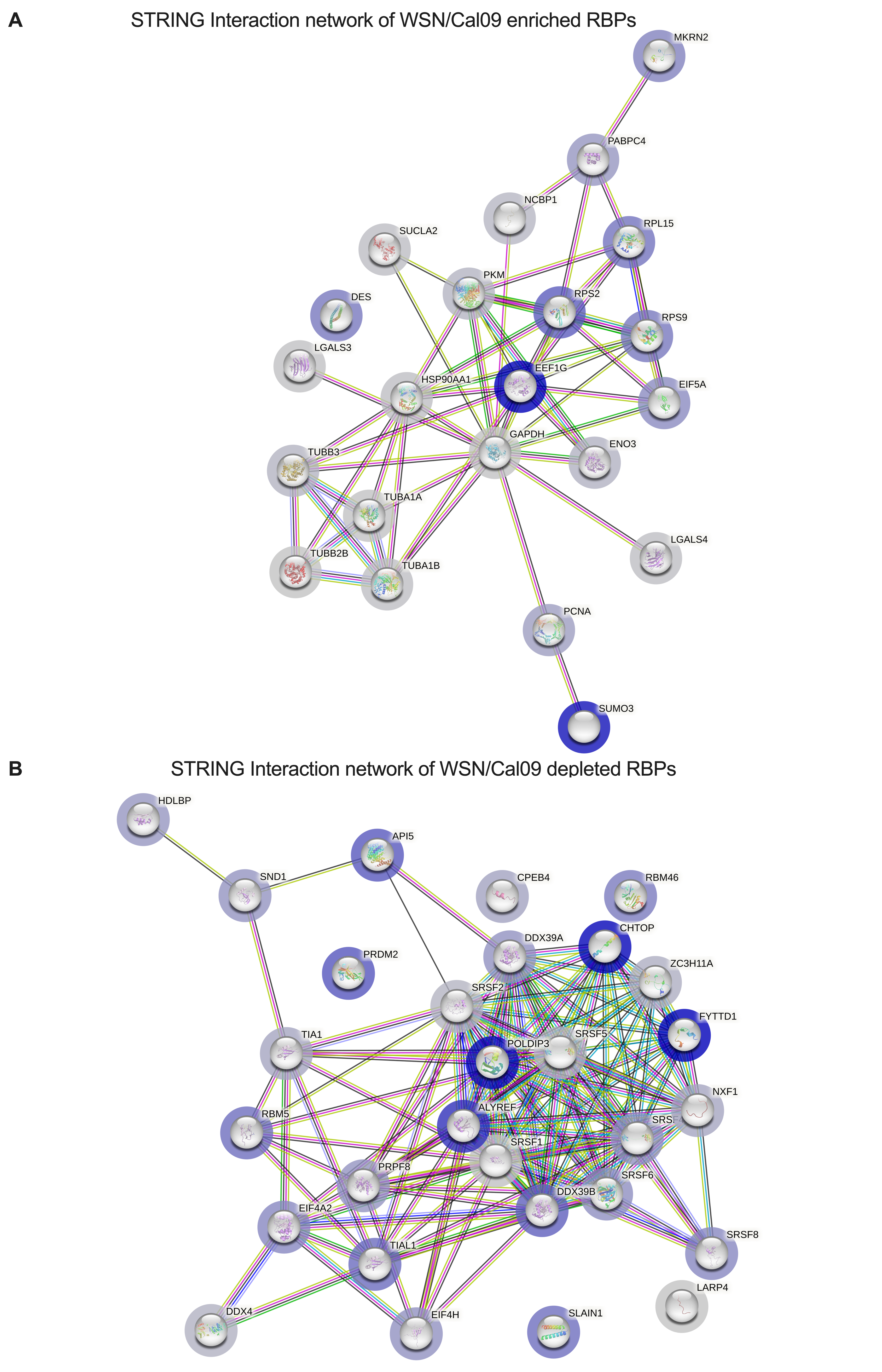

Supplement: S1 Fig — (A) STRING analysis of the RBPs enriched in the RIC samples of WSN and Cal09 infected A549 cells. (B) STRING analysis of the RBPs depleted in the RIC samples of WSN and Cal09 infected A549 cells. (TIF) [file ppat.1012231.s001.tif]

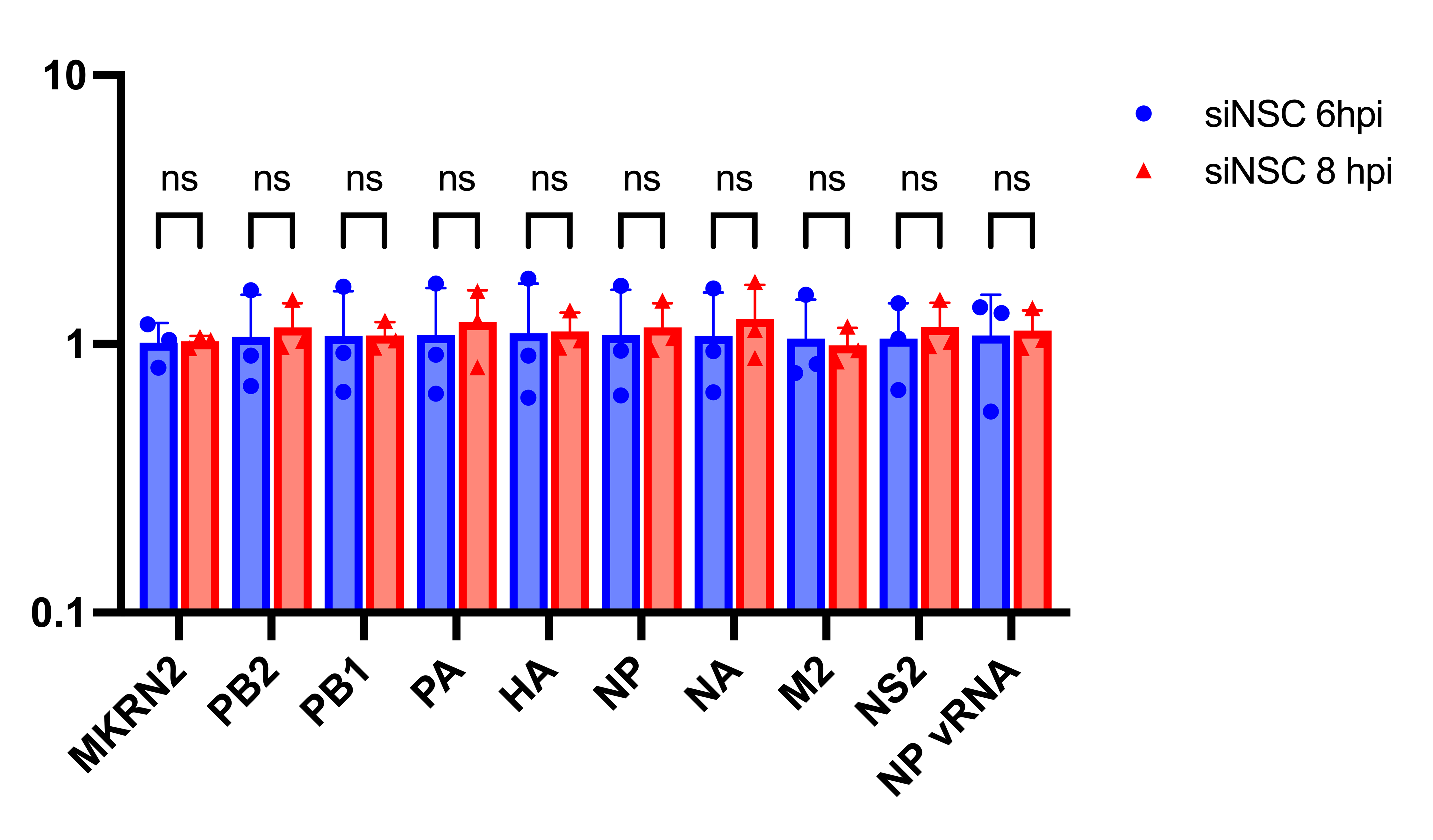

Supplement: S2 Fig — This was to identify whether steady state levels of these transcripts differed between these 2 timepoints. This uses the same values as have been used in Fig 5E and 5F, but in this instance the 8 hpi levels were normalised to 6 hpi. (TIF) [file ppat.1012231.s002.tif]

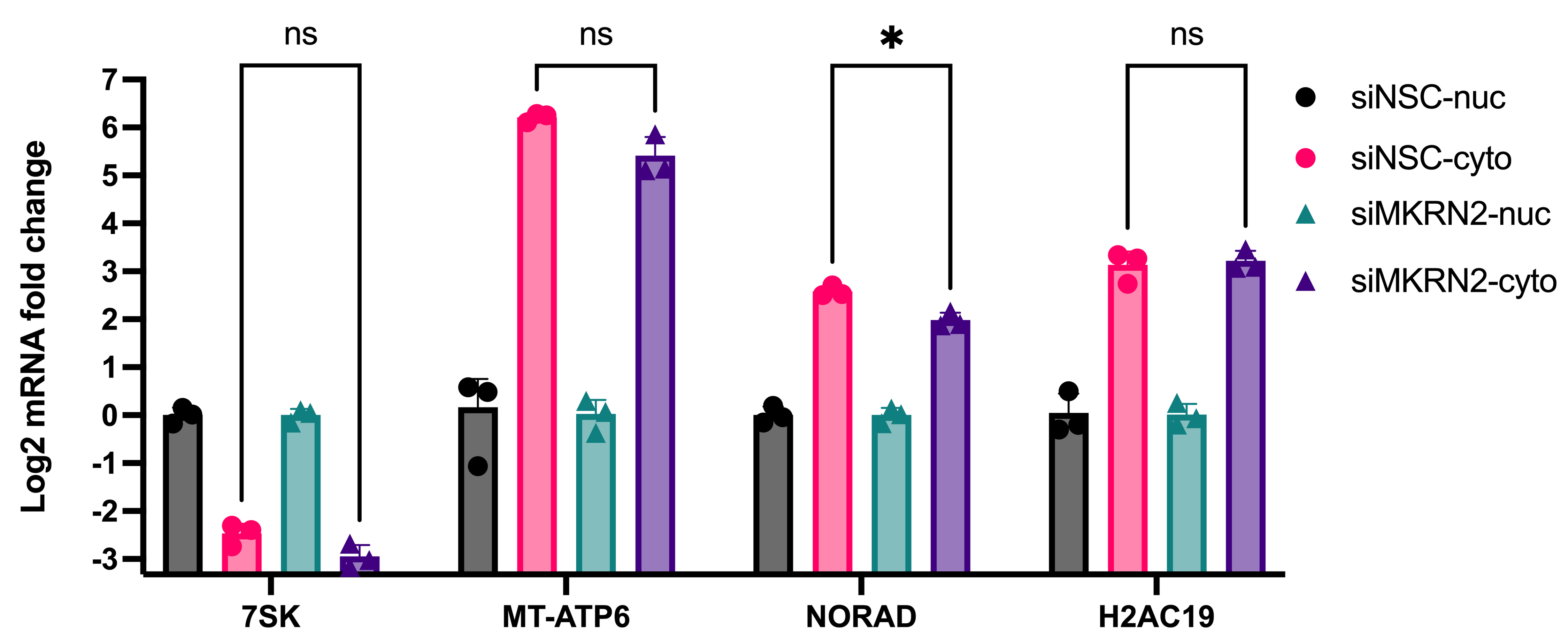

Supplement: S3 Fig — These are the same samples used to generate Fig 6F. The levels of 7SK, MT-ATP6, NORAD and H2AC19 were quantified with the cytoplasmic transcript level normalised to that of the corresponding nuclear fraction. 7SK and MT-ATP6 were used to confirm adequate nuclear and cytoplasmic fractions, respectively, with 7SK demonstrating a clear nuclear localisation, while MT-ATP6 was found to be predominantly cytoplasmic. NORAD and H2AC19 were 2 representative unspliced host mRNA used to determine whether unspliced mRNA localisation was altered upon MKRN2 depletion. (TIF) [file ppat.1012231.s003.tif]
